# The Incidence of Cystic Fibrosis in the Central Region of Anatolia in Turkey Between 2015 and 2016

**DOI:** 10.4274/balkanmedj.galenos.2018.2018.1332

**Published:** 2019-05-10

**Authors:** Melih Hangül, Sevgi Pekcan, Mehmet Köse, Deniz Acıcan, Tuba Esra Şahlar, Murat Erdoğan, Mustafa Kendirci, Deniz Güney, Hasan Öznavruz, Osman Demir, Ömür Ercan, Fatma Göçlü

**Affiliations:** 1 Department of Pediatrics, Division of Pediatric Pulmonology, Erciyes University School of Medicine, Kayseri, Turkey; 2 Department of Pediatrics, Division of Pediatric Pulmonology, Necmettin Erbakan University Meram Faculty of Medicine, Konya, Turkey; 3 Department of Child and Adolescent, Public Health General Directorate, Ankara, Turkey; 4 Konya Provincial Public Health Directorate, Konya, Turkey; 5 Clinic of Genetics, Kayseri City Hospital, Kayseri, Turkey; 6 Departman of Pediatrics, Division of Pediatric Nutrition and Metabolism, Erciyes University School of Medicine, Kayseri, Turkey; 7 Kayseri Provincial Public Health Directorate, Kayseri, Turkey

**Keywords:** Cystic fibrosis, incidence, middle Anatolia, newborn screening, Turkey

## Abstract

**Background:**

Cystic fibrosis is the most common metabolic chronic disease among European Caucasian children. Cystic fibrosis incidence in Northern Europeans countries is approximately 1 in 3000 births while the worldwide prevalence varies considerably.

**Aims:**

To determine the incidence of cystic fibrosis in the central region of Anatolia in Turkey using the newborn screening program data.

**Study Design:**

Cross-sectional study.

**Methods:**

We used the records of the newborn screening program which is implemented by the Konya and Kayseri Provincial Health Directories. Between January 2015 and December 2016, there were a total of 119006 live births in Konya and Kayseri. The newborn screening test was applied to all these babies.

**Results:**

During this period, there were 22 live born babies diagnosed with cystic fibrosis in Konya with an incidence of 2.9 per 10000 live births and 13 live born babies diagnosed with cystic fibrosis in Kayseri with an incidence of 2.8 per 10000 live births. In genetic of 30 patients, fifteen patients were homozygous, and 15 patients were a compound heterozygote. Twenty-one different gene variants were detected and the most common mutation was F508del (17/30).

**Conclusion:**

We found the incidence of cystic fibrosis in central Anatolia similar to northern European countries.

Cystic fibrosis (CF) is the most common chronic disease in Caucasian children. CF is an autosomal recessive disorder and the most common mutation of CF is DF508 (1,2). The gene encoding the CF transmembrane conductance regulator protein is located on chromosome 7 (3). CF is clinically characterized by the obstruction and infection of the airways and gastrointestinal tract (1). The incidence of CF varies worldwide (4). In Northern European countries, CF incidence is 1 in 3000 births (5), while the frequency of CF in Turkey is not clearly known. It is possible to obtain data on the actual frequency of the disease in Turkey by using the national newborn screening (NBs) program, which is conducted by the Child and Adolescent Department of the Public Health General Directory. In this paper, we describe the method and result of the CF incidence using data of the NBs program in their first 2 years. We use data of two large cities in Turkey, determine the live-birth incidence, demographic, and clinical features of CF.

## MATERIALS AND METHODS

This study was approved by the Erciyes University ethics committee (number: 2016/642) and informed written consent was obtained from the patients’ parents. The publication of the national screening program data was approved by the Public Health General Directory, Child, and Adolescent Department.

In this retrospective cross-sectional study, we evaluated the records of the newborn NBs program, between January 1, 2015, and December 31, 2016, for CF. The program is run by the Konya and Kayseri provincial health care services. We assessed demographic data, clinical features, symptoms at first admission, and genetic analysis results. According to these records, we evaluated patients who were immunoreactive trypsinogen (IRT) positive. Babies with their first IRT blood values >90 ng/mL were evaluated a second time. If the second IRT blood values >70 ng/mL, the babies were directed to CF centers (Necmettin Erbakan University, School of Medicine and Erciyes University, School of Medicine) where sweat tests were performed. These two university hospitals are the only centers specializing in CF in these cities.

The diagnosis of CF is based on the sweat chloride levels (>60 mmol/L), two identified CF mutations, and characteristic CF symptoms (1,6). We also used the CF records of these two universities between January 1, 2015, and December 31, 2016, to find CF patients diagnosed without positive NBs results.

The genetic analysis of the patients begun obtaining genomic DNA from blood using the MagPurix Blood DNA Extraction Kit 200. A CF transmembrane conductance regulator gene library was constructed using the Nextflex Cystic Fibrosis Amplicon Panel (Austin, Texas) according to the manufacturer's instructions. This kit contains 61 double primers and allows the analysis of 27 exon and exon-intron connections of the CF transmembrane conductance regulator gene. The kit covers all the pathogenic variants in the NCBI ClinVar database. Sequencing was performed with the MiSeq Illumina (San Diego, California) platform. The obtained data were evaluated with Seq Genomize (İstanbul, Turkey) and Integrative genomics viewer software. The mutation names, type, genomic region, genomic localization, and legacy protein nomenclature were determined according to the nomenclature of the Human Genome Variation Society.

### Statistical analysis

Birth incidence was calculated by dividing the number of new cases of CF by the number of live births.

## RESULTS

**Figure 1:**
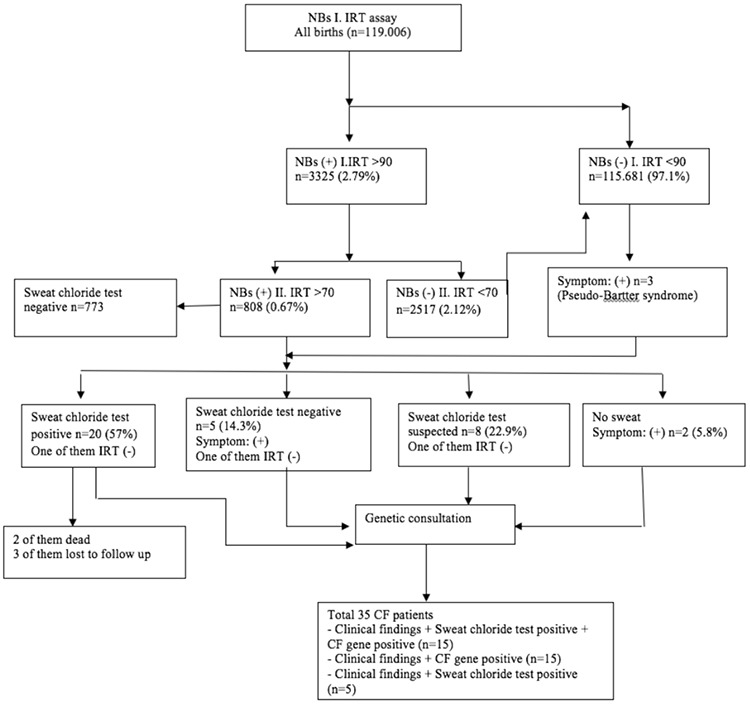
Cystic fibrosis newborn screening algorithm and results overview. CF: cystic fibrosis; NBs: newborn screening; IRT: immunoreactive trypsinogen

Between 2015 and 2016, there were 119.006 live births in the Konya and Kayseri provinces. NBs test was performed on all these babies. At the first IRT screening, 3.325 newborn babies were found with CF. After the second examination, only 808 babies remained and were referred to CF centers for further evaluation. All the babies were given a sweat test. Twenty (57%) of them had a sweat test result >60 mmol/L that was consistent with CF, eight (22.9%) of them were found to have a suspicious sweat test (30-59 mmol/L), and 5 (14.3%) of them were negative (<30 mmol/L). We could not perform the sweat test in two patients (5.8%). Although the IRT was negative, three patients were diagnosed as having CF due to findings of Pseudo-Bartter’s syndrome and genetic results. A total of 35 patients were diagnosed with CF using newborn screening, sweat test results, clinical findings, and genetic screening (Figure 1). Two patients who underwent surgery for meconium ileus died before their follow-up. Three patients did not come to the hospital for follow-up. Therefore, genetic analysis was performed only in 30 patients. Fifteen patients were homozygous, and 15 patients were a compound heterozygote. Twenty-one different gene variants were detected in 30 patients. The most common mutation in our patients was F508del (17/30), followed by G85E, N1303K, G542X, and W1282X (Table 1).

Demographic and clinical characteristics of patients were as follows: 45.8% of the patients were female and 54.2% were male. Patients were diagnosed at an average of 45 (minimum: 3; maximum: 300) days. At the time of diagnosis, the most common symptom was Pseudo-Bartter syndrome (36.9%). Thirteen (34.3%) patients were asymptomatic, and 22 (65.7%) patients had one or two symptoms (Table 2).

There were 10 live births patients with CF (from 37.567 live births in Konya) giving an incidence of 2.6 per 10.000 (i.e. 1/3.756) live births in 2015 and 12 live births patients with CF (from 35.962 live births in Konya) giving an incidence of 3.3 per 10 000 (i.e. 1/2.996) live births in 2016. There were eight live births patients with CF (from 23.077 live births in Kayseri) giving an incidence of 3.4 per 10.000 (i.e. 1/2.884) live births in 2015 and 5 live births patients with CF (from 22.400 live births in Kayseri) giving an incidence of 2.2 per 10.000 (i.e. 1/4480) live births in 2016. Consequently, the records obtained over the two-year period were evaluated and there were 35 live births patients with CF (from 119.006 live births in Konya and Kayseri) giving an incidence of 2.9 per 10.000 (i.e. 1/3.400) live births between January 1, 2015, and December 31, 2016.

## DISCUSSION

In this study, we found the incidence of CF to be 1 in 3.400 live births in the central Anatolia region. This study is the first Turkish incidence study based on NBs. The NBs program, which started on January 1, 2015, makes possible to recognize the CF patients earlier. Many studies have shown that early diagnosis of CF has an important role in increasing the average lifespan. because these patients will have better nutritional status, growth parameters, and healthier lungs than patients diagnosed later (7,8).

The NBs program facilitated a better record of patients and obtaining information about the frequency of the disease. Previously, the frequency of CF in Turkey was not clearly known and we did not have enough knowledge about the incidence of CF. Only a small study in 1973, before the NBs programs and with limited data, found the incidence of CF to be 1 in 3.000 live births in Turkey (9). Our results were similar to this study. The differences between the study by Gürson et al. (9) and our study was that their study was not multicentric, it only included patients from İstanbul. Moreover, the first aim of Gürson et al. (9) was to compare the sweat test method and the neutron activation method. In their study, 6061 patients were screened to investigate the efficacy of these tests for CF, and they found two patients diagnosed with CF. They calculated CF incidence to be 1/3000 (9). Other than this study, there is no other report showing the incidence of CF. The first aim of our study was to determine the incidence of CF in the central region of Anatolia in Turkey.

The highest CF incidence is seen in Northern European countries with 1/3.000 live births. In the United States, the disease occurs in roughly 1 in 3.000 white Americans, 1 in 4.000-10.000 in Hispanics, and 1 in 15.000-20.000 in African Americans (10). In Africa and Asia CF is very rare. In Japan, the incidence is only 1/350.000 live births (11). Konya and Kayseri are the oldest settlements in Turkey and are in the middle of the Anatolian region. Due to its location, this region is influenced by both Western and Eastern cultures. These cities have more than 3.500.000 inhabitants and are leading industrial and trade centers, which receive emigrants from other parts of Turkey. These two cities have the potential to reflect the entire middle Anatolia. Our results showed that CF in middle Anatolia is as prevalent as in Northern European countries.

In this study, eight patient’s sweat test was suspicious and five were negative. In Turkey’s CF centers, infants whose second IRT results are positive, even though they had a negative or suspicious sweat test, are followed clinically. Patients with a negative and suspected sweat test have a second test one month later. Therefore, the false negative results that may be caused by the sweat test are reduced. When the sweat test results are evaluated, sometimes-false negative results can be obtained (12). In addition to biological variability and some CF transmembrane conductance regulator mutations, false negative sweat testing may occur due to technical errors (13). For this reason; we think that patients with positive IRT results should be monitored for a certain period even though the sweat test is negative.

The IRT is a screening test, not a diagnostic test, which can lead to false negative results. Despite this, screening tests have great significance in terms of early disease identification. In our study, 3 (0.2%) patients, who have CF symptoms, were diagnosed with CF even though their IRT was negative. We reviewed the results of other countries NBs tests, the false negative rate reported in Australia is 5% (14), 1.7% in the United States, and 3.4% in France (8,15). Even though the NBs test can have false negative results, they can identify most of the patients. In false-positive individuals, serum IRT is reduced much more rapidly than in children with CF, for this reason, a higher second IRT has a stronger positive predictive value (16). Interpretation of IRT tests is complex, due to confounding factors such as age-related declines in IRT blood levels, prematurity, perinatal stress, fecal contamination of blood spots, trisomy 13 and 18, other serious congenital abnormalities, renal failure, bowel atresia, nephrogenic diabetes insipidus, congenital infections, and neonatal blood transfusions (17,18,19). A false-positive IRT value could lead the patient over follow-up and increases the anxiety in the families (20,21). This is a disadvantage of the NBs test. New studies can be performed to reduce false-positive results without reducing the sensitivity of the test.

The median time for diagnosing infants was 45 days. The earliest diagnosis was 3 days and the latest diagnosis was 300 days. The infants who were diagnosed the latest had negative NBs results. All of them had Pseudo-Bartter’s syndrome and were diagnosed with CF according to the genetic results. At the time of diagnosis, 36.9% of the patients had a Pseudo-Bartter syndrome and 34.3% of the patients were asymptomatic while other patients had one or more CF-related symptoms. The patients who were asymptomatic could not be diagnosed previously and were recently diagnosed by the NBs test. Early diagnosis of CF has an important role in increasing the average lifespan and this shows us the importance of the screening test.

We requested genetic counseling for 35 patients diagnosed with CF. However, 5 (14.3%) patients were lost during follow-up. CF mutation was detected in 30 (85.7%) patients. The frequency of the most common mutation, DF508, was 40% in the middle Anatolia population. Seven of these patients were homozygous and 10 were heterozygous. Other common mutations in our study were G85E (8.3%), N130K (6.6%), G42X (5%), and W128X (5%) (Table 2). In the study by Yilmaz et al. (22), the frequency of the DF508 mutation was 28.4% in Turkey and in a study by Onay et al. (23) the frequency was 25%. In our study results, the frequency of DF508 (40%) was higher than these studies. Sample numbers were different, and regional ethnic differences may explain this situation in Turkey. Turkey has many different ethnic groups due to historical migration routes. Several studies have shown that the frequency of the DF508 mutation differs in different countries: with frequencies of 86.9% in Denmark (24), 73% in Scotland (25), 55% in Italy (26), 54% in Greece (27), 17.8%, in Iran (28), and 17.9% in Tunisia (29). Our study result was lower than European countries but higher than eastern countries. In our study, 21 different mutations were detected in 30 patients. This data confirms the considerable molecular heterogeneity of CF among the Turkish population. To illustrate Turkey’s genetic structure better, further studies with larger samples are necessary.

One of the limitations of this study is the absence of all Turkey regions NBS program data. Another limitation is the lack of diagnosis in newborn babies who died without being screened. This problem affects studies focusing on incidence, including ours.

In conclusion, this is the first study to determine the incidence of CF in middle Anatolia and shows the incidence of CF is similar to Northern European countries.

## Figures and Tables

**Table 1:**
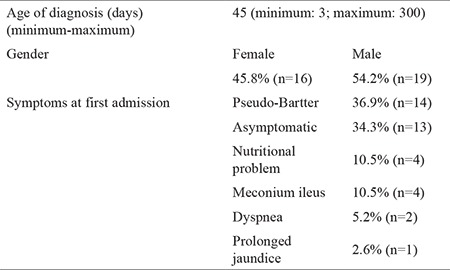
Demographic and clinical features of cystic fibrosis patients

**Table 2:**
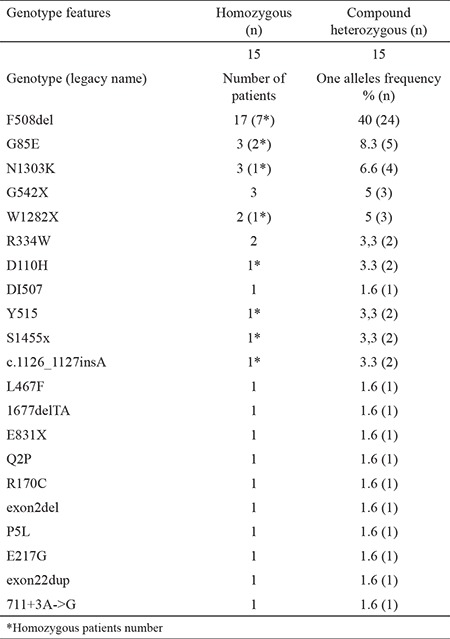
Genetic results of children with cystic fibrosis mutations
